# Therapy de‐escalation in paediatric patients with inflammatory bowel disease in remission: Data analysis from the CEDATA registry

**DOI:** 10.1002/jpn3.70297

**Published:** 2025-12-12

**Authors:** Sila Cekin, Christoph Huenseler, Serdar Cantez, Ilse Broekaert, Jan de Laffolie, CEDATA‐GPGE® Study Group

**Affiliations:** ^1^ Department of Paediatrics, Faculty of Medicine and University Hospital Cologne University of Cologne Cologne Germany; ^2^ Department of General Paediatrics and Neonatology, Pediatric Gastroenterology University of Giessen Giessen Germany

**Keywords:** biologic, combination therapy, immunomodulator, prognostic factors, withdrawal

## Abstract

**Objectives:**

There are no standardized criteria about stepping down from combination therapy (immunomodulator and tumor necrosis factor (TNF)‐alpha‐inhibitors) in children with inflammatory bowel disease (IBD) to reduce risk for side effects. Our aim was to describe how de‐escalation has been performed in a large paediatric cohort and to find prognostic factors for therapy de‐escalation without the need for therapy adjustments post‐de‐escalation.

**Methods:**

Real‐world data from CEDATA‐GPGE, a German‐Austrian registry for paediatric IBD patients, from 2004 to 2023, were analyzed. Patients not requiring therapy adjustments post‐de‐escalation and patients requiring therapy adjustments after de‐escalation were compared, and prognostic factors were identified.

**Results:**

Two hundred and thirty out of 6248 registered patients received combination therapy for at least 6 months. In 64 patients therapy adjustment was not required after de‐escalation. Crohn's disease (CD) patients, younger patients, and patients with positive modified predictors of poor outcome were significantly more often on combination therapy. Regarding de‐escalation, CD patients were more often successfully de‐escalated than ulcerative colitis (UC) and IBD‐unclassified patients. UC patients with a less severe disease manifestation (Paris classification E1 or E2) were de‐escalated more successfully than those with more extensive disease (E3 or E4). De‐escalation to monotherapy with a biologic led to a more successful de‐escalation than de‐escalation to immunomodulator monotherapy or stopping both biologic and immunomodulator.

**Conclusions:**

De‐escalation is more likely successful in patients with CD, and de‐escalating combination therapy to monotherapy with a TNF‐alpha‐inhibitor is more advantageous.

## Abstract (highlights)

**What is Known:**

- Combination therapy is recommended for children with inflammatory bowel disease, but no evidence‐based guidelines for therapy de‐escalation in patients in remission exist.
- The decision of who, what, and when to de‐escalate is one of the most important yet most challenging decisions for both the patient and clinician.

**What is New:**

- Our study identifies considerable prognostic factors that can help in the decision‐making process.
- Combination therapy is more likely successful in children with Crohn's Disease, patients that are de‐escalated to monotherapy with a biologic, and ulcerative colitis patients with less severe disease manifestation (Paris classification E1 or E2).

## Graphical abstract

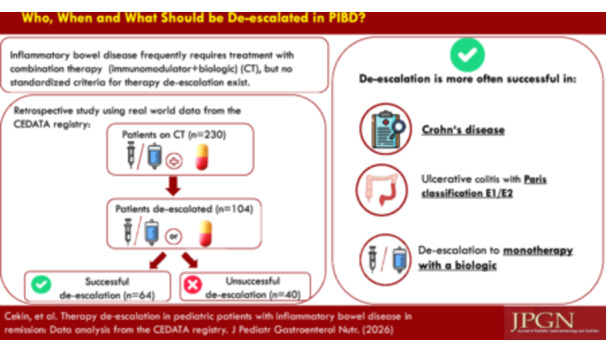

## 1 INTRODUCTION

Inflammatory bowel diseases (IBDs), including Crohn's disease (CD), ulcerative colitis (UC), and IBD‐unclassified (IBD‐U), can significantly impair quality of life, especially at a young age.^1^ Children with IBD, due to a more active and extensive disease course, depend on more intensive therapy compared to adult patients.^2^ A combination therapy consisting of an immunomodulator (such as thiopurines) and a biologic (such as tumor necrosis factor‐alpha inhibitors [TNF‐alpha‐inhibitors]) is an option for children with IBD.^3^ There are, however, no guidelines for subsequent de‐escalation in patients in prolonged remission.^4^

Combination therapy carries the risk of side effects such as infections, but also tumors of the lymphatic system, for example, hepatosplenic T‐cell lymphoma, or dermatologic malignancies, such as melanotic and nonmelanotic skin cancer, which in some cases can develop several years after discontinuation of therapy.^4^, ^5^, ^6^ Guidance on when, how, and in whom to de‐escalate without the need for therapy adjustment post‐de‐escalation could facilitate clinical decision making and improve outcome. Furthermore, de‐escalation could reduce therapy costs.^7^

The aim of this retrospective analysis of prospectively collected data was to describe how de‐escalation has been performed in the real world and to find prognostic factors for successful de‐escalation meaning patients not requiring therapy adjustments within 12 months after de‐escalation versus those requiring therapy adjustment after withdrawal.

## 2 METHODS

### 2.1 Ethics statement

CEDATA‐GPGE® is a multicentre registry with approximately 90 participating clinics in Germany and Austria and is approved by the Ethics Committee of the University Hospital Giessen. The research was conducted in accordance with ethical standards outlined in the Declaration of Helsinki.

### 2.2 Study design

Real‐world data from the CEDATA‐GPGE®‐registry from January 2004 to January 2023 were examined. CEDATA‐GPGE® is a prospective, multicentre registry with approximately 90 participating clinics in Germany and Austria. Data are pseudonymously entered in a safe web‐based platform at least every 6 months during routine visits.^8^

Inclusion criteria were age below 18 years of age, a diagnosis of IBD according to the Porto criteria, and combination therapy for at least 6 months.^9^ Combination therapy was defined as a therapy consisting of infliximab (IFX) and thiopurines (azathioprine [AZA]), IFX and methotrexate (MTX), and adalimumab (ADA) and thiopurines, patients with other combination therapies were not included due to low patient volume. For a reliable outcome assessment and the prevention of selection‐bias, we chose a follow‐up of at least four visits for all patients. The minimal follow‐up after de‐escalation was 12 months.

Due to a different method of data entry, patients registered before December 2016 were excluded if the interval between initial reporting and first documentation was more than 2 weeks. Patients who did not register within 3 months of diagnosis were also excluded from the study. By excluding those patients with delayed data collection, we tried to prevent recall‐bias and thus increase the quality of our study.

De‐escalation was defined as:

‐ dose reduction of either the immunomodulator or biologic
‐ complete discontinuation of the immunomodulator or biologic or both.

Patients who did not require therapy intensification within 12 months after de‐escalation were considered as successfully de‐escalated. Patients requiring therapy intensification within 12 months after de‐escalation were considered as unsuccessfully de‐escalated. Therapy escalation was defined as:

‐ restarting previously discontinued medication
‐ increasing the dose of the continued medication
‐ start of a drug from the same class (biologic or immunomodulator) that had been previously discontinued
‐ starting corticosteroids
‐ starting nutritional therapy for CD.

Both groups (successful and unsuccessful de‐escalation) were compared regarding basic characteristics such as diagnosis, age at diagnosis, gender, and modified predictors of poor outcome (POPO) criteria including five of the seven POPO criteria (POPO‐2: persistent disease, POPO‐3: extensive disease, POPO‐4: severe growth retardation, POPO‐6: stricturing/penetrating disease, POPO‐7: perianal disease), as well as disease activity scores: short pediatric Crohn's disease activity index (sPCDAI) at diagnosis and pediatric ulcerative colitis disease activity index (PUCAI) at diagnosis. ^10^, ^11^, ^12^, ^13^ Treatment modalities such as therapy latency (time from first diagnosis to begin of combination therapy) and the duration of combination therapy were compared between the two groups. The following variables were also compared between the two groups by comparing the last recorded value within 3 months before de‐escalation: Paris classification, extraintestinal manifestations (EIMs), fecal calprotectin levels, AZA dose, IFX dose, IFX levels, and presence of IFX‐antibodies.

### 2.3 Statistical analysis

Statistical analysis was performed using SPSS version 29.0.0.0 (IBM corp©). After conducting tests of normality (Kolmogorov–Smirnova and Shapiro–Wilk) to determine which of the potential influencing factors were normally distributed, the Mann–Whitney–*U*‐test was used to investigate significant differences regarding the non‐normally distributed variables between the groups. The non‐normally distributed variables included: age at diagnosis, latency between diagnosis and start of combination therapy and duration of combination therapy. The following variables were also nonnormally distributed and of those the last documented value within the last 3 months before de‐escalation were included: AZA dose, IFX dose, IFX and calprotectin levels. sPCDAI at diagnosis and PUCAI at diagnosis were normally distributed and therefore compared with *t*‐test for independent values between the groups. To compare nominal variables the chi‐square test was used. The *p*‐value for statistical significance was defined as <0.05.

## 3 RESULTS

**Figure 1:**
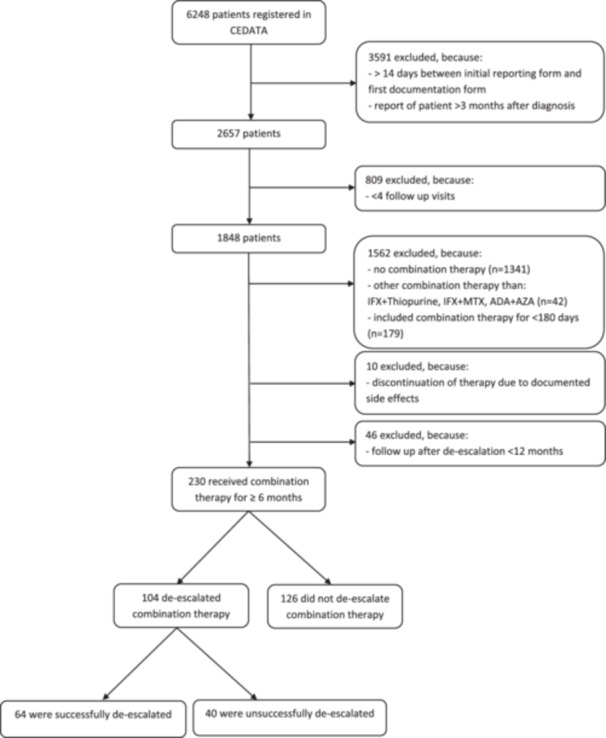
Flow chart study flow, depicting the application of in‐ and exclusion criteria, the resulting group sizes, and the number of successfully and un‐successfully deescalated patients.

In the CEDATA‐GPGE‐registry, 6248 patients were registered and 6018 were excluded due to various reasons: delayed reporting to the data registry was seen in 3591 patients and 1562 patients did not receive a combination therapy included in the study. Early discontinuation of combination therapy due to side effects was seen in 10 patients, and 855 patients did not meet the minimum follow‐up time requirement (Figure 1).

### 3.1 Combination therapy versus no combination therapy

After application of the exclusion criteria, 230 patients received combination therapy. One hundred and sixty‐eight patients received IFX and AZA (73%), 39 IFX and MTX (17%), and 23 ADA and AZA (10%). Of the 230 patients 76.5% were diagnosed with CD, 19.1% with UC and 4.3% with IBD‐U. Median time between diagnosis and start of combination therapy was 355 days [113.5; 975]. Median duration of combination therapy was 453.5 days [299; 849.5] (Table 1).

**Table 1:** Demographic and clinical characteristics of patients that received combination therapy versus no combination therapy.

	Combination therapy (*n* = 230)	No combination therapy (*n* = 1562)	*p*
Sex: male, *n* (%)	143 (62.2)	880 (56.3)	0.095
Age at diagnosis in years, median (range)	11.9 (9.5–13.8)	12.8 (10.4–14.7)	<0.001
Diagnosis, *n* (%)			<0.001
CD	176 (76.5)	916 (58.6)	<0.001
UC	44 (19.1)	564 (36.1)	<0.001
IBD‐U	10 (4.3)	82 (5.2)	0.563
Positive for at least one modified predictor of poor outcome, *n* (%)	140 (79.5)	424 (27.1)	<0.001

Abbreviations: CD, Crohn's disease; IBD‐U, colitis indeterminate; UC, ulcerative colitis.

### 3.2 De‐escalation versus no de‐escalation

Of the 104 patients that were de‐escalated, 76% had CD, 21.2% UC, and 2.9% IBD‐U. Median age at diagnosis was 11.6 years (9.3; 13.5). The latency from diagnosis to the initiation of combination therapy was a median of 372 days (126.8; 984), and the median duration of combination therapy was 377.5 days (248; 607.3) (Table 2). Seventy‐six patients (73.1%) de‐escalated from AZA and IFX, 18 patients (17.3%) de‐escalated from MTX and IFX, 10 patients (9.6%) de‐escalated from AZA and ADA. In most cases, de‐escalation was done abruptly, while in four de‐escalated patients de‐escalation was done gradually, meaning they experienced a quantitative reduction instead of the abrupt ending of the medication.

**Table 2:** Demographic and clinical characteristics of patients that were de‐escalated versus no de‐escalation.

	De‐escalation (*n* = 104)	No de‐escalation (*n* = 126)	*p*
Sex: male, *n* (%)	65 (62.5)	78 (61.9)	0.845
Diagnosis, *n* (%)			0.515
CD	79 (76)	97 (77)	0.856
UC	22 (21.2)	22 (17.5)	0.478
IBD‐U	3 (2.9)	7 (5.6)	0.323
Positive for at least one modified predictor of poor outcome, *n* (%)	67 (64.4)	73 (58)	0.101
sPCDAI, median (range)	35 (23‐55)	45 (25‐55)	0.132
PUCAI, median (range)	35 (15–60)	45 (35–63)	0.180

Abbreviations: CD, Crohn's disease; IBD‐U, colitis indeterminate; PUCAI, pediatric ulcerative colitis activity index; sPCDAI, short pediatric Crohn's disease activity index; UC, ulcerative colitis.

### 3.3 Patients not requiring therapy adjustments after de‐escalation versus patients requiring therapy adjustments after de‐escalation

Sixty‐four patients did not require therapy adjustments after de‐escalation. Of those 64 patients, 55 had CD (85.9%), eight had UC (12.5%), and one patient had IBD‐U (1.6%). Forty‐seven of the CD patients (73.4%) had at least one positive modified POPO criteria.^9^ There was a median latency from diagnosis to the start of combination therapy of 358.5 days (130.8; 1019.3), with a median duration of combination therapy of 418.5 days (249.8; 628) (Table 3).

**Table 3:** Demographic and clinical characteristics of patients that were successfully de‐escalated versus unsuccessfully de‐escalated.

	Patients de‐escalated without requiring therapy adjustments post‐de‐escalation	Patients de‐escalated requiring therapy adjustments post‐de‐escalation	*p*
	(*n* = 64)	(*n* = 40)	
Sex: male, *n* (%)	24 (37.5)	25 (62.5)	1
Age at diagnosis in years, median (range)	11.35 (9.2–13.4)	12.42 (9.5–13.8)	0.343
Diagnosis, *n* (%)			0.011
CD	55 (85.9)	24 (60)	0.003
UC	8 (12.5)	14 (35)	0.006
IBD‐U	1 (1.6)	2 (5)	0.488
sPCDAI, mean (standard deviation)	37.6 (18)	34 (22.8)	0.52
PUCAI, mean (standard deviation)	25 (26.1)	43.6 (18.7)	0.086
Within 3 months before de‐escalation:			
Calprotectin in µg/g, median (range)	98 (26–358)	162 (86–362)	0.219
Cut‐off 250 µg/g, *n* (%)	16 (34)	10 (41.2)	0.53
Cut‐off 350 µg/g, *n* (%)	13 (27.7)	7 (29.2)	0.89
Cut‐off 500 µg/g, *n* (%)	11 (23.4)	5 (20.8)	0.8
EIM positive, *n* (%)	10 (15.6)	4 (10)	0.414
AZA dose in mg, median (range)	100 (75–100)	100 (75–125)	0.464
AZA dose in mg/kg, median (range)	1.7 (1.4–2.0)	1.7 (1.4–2.2)	0.499
IFX dose in mg, median (range)	300 (200–400)	300 (300–400)	0.336
IFX dose in mg/kg, median (range)	5.7 (4.8–8.0)	5.9 (4.9–8.0)	0.595
IFX levels in µg/mL, median (range)	4 (1.3–7.7)	4.5 (2.9–7.5)	0.599
De‐escalation to			0.041
AZA monotherapy, *n* (%)	16 (25)	12 (30)	0.576
ADA monotherapy, *n* (%)	11 (17.2)	1 (2.5)	0.023
IFX monotherapy, *n* (%)	33 (51.6)	20 (50)	0.877
MTX monotherapy, *n* (%)	3 (4.7)	2 (5)	0.942
Nothing, *n* (%)^a^	1 (1.6)	5 (12.5)	0.02

Abbreviations: ADA, adalimumab; AZA, azathioprine; CD, Crohn's disease; EIM, extraintestinal manifestations; IBD‐U, colitis indeterminate; IFX, infliximab; MTX, methotrexate; PUCAI, pediatric ulcerative colitis activity index; sPCDAI, short pediatric Crohn's disease activity index, UC, ulcerative colitis.

^a^ Nothing = no immunomodulator and no biologic.

Comparing patients who did not need therapy adjustments after de‐escalation with the group that required therapy adjustments after de‐escalation, CD patients required therapy adjustments less often after de‐escalation than UC and IBD‐U patients (69.6% vs. 36.4% vs. 33.3%, *p* = 0.011). Among UC patients, those with less severe disease (Paris classification E1 or E2) were more likely to be successfully de‐escalated than those with more extensive disease (Paris classification E3 or E4) (100% vs. 18.2%, *p* = 0.002). Furthermore, patients de‐escalated to monotherapy with a biologic were more often successfully de‐escalated compared to de‐escalation to immunomodulator monotherapy or stopping both biologic and immunomodulator therapies (67.7% vs. 42.4% vs.16.7%, *p* < 0.041) (Table 3).

Of the 40 patients that needed therapy escalation after de‐escalation, 18 patients (45%) re‐started the same combination therapy, nine (22,5%) began another combination therapy than they initialy started with, eight (20%) started steroids, four (10%) increased the dose of the continued medication, and one CD patient (2,5%) patient started with nutritional therapy.

## 4 DISCUSSION

Candidates for successful de‐escalation from combination therapy in remission need to be identified to minimize long‐term risks either from therapy.^14^, ^15^, ^16^ However, evidence‐based data on de‐escalation of therapy in children with IBD are lacking. Analysis of real‐world data from the CEDATA‐GPGE® registry, a large German‐Austrian prospective multicentre registry of paediatric IBD patients identified prognostic factors for successful de‐escalation, which will help clinicians with decision making and will prevent unnecessary therapy.^8^, ^17^

In our study, we found that de‐escalation of CD patients, UC patients with Paris‐classification E1 or E2 (compared to E3 or E4), and reduction to monotherapy with a biologic (compared to an immunomodulator or stopping both) were significantly less likely to require therapy adjustment post‐de‐escalation.

Gisbert et al. performed a meta‐analysis on anti‐TNF‐withdrawal after combination therapy with IFX and ADA including 27 studies, of which two were paediatric. After step‐down to immunomodulator monotherapy, the overall relapse‐risk was 44% for CD and 38% for UC patients. Of the patients in our study that were de‐escalated to an immunomodulator, the overall relapse‐risk was 35% for CD patients (7/20) and 62,5% for UC patients (5/8). Both studies show that CD patients are more likely to step‐down from treatment without relapsing than UC patients, while the figures from our study show this more clearly. However, the results are comparable to a limited extent, as most of the studies included in Gisbert et al. meta‐analysis are adult studies. Also, the size of this subgroup in our study is rather small, nevertheless a trend can be seen.^18^

Kierkus et al. conducted the only randomized control trial (RCT) comparing combination therapy and monotherapy in paediatric CD. Eighty‐four children in remission on either IFX and AZA or IFX and MTX were randomized to either continue combination therapy or de‐escalate to IFX‐monotherapy. They found that de‐escalating to IFX‐monotherapy was as effective as continuing combination therapy with similar relapse rates of about 30%.^19^

El‐Matary et al. conducted the first paediatric retrospective study assessing immunomodulator withdrawal after combination therapy with anti‐TNF and reported a relapse rate of only 10.5% patients during the 2 years of follow‐up.^15^ Several adult studies further support this observation. Meta‐analysis by Dohos et al. and Chalhoub et al. investigated the outcomes of immunomodulator withdrawal from ADA‐ combination therapy in adult CD patients and found no increased risk of relapse after thiopurine withdrawal.^20^, ^21^, ^22^ Similarly, RCTs by Roblin et al. and Van Assche et al. found no significant difference in relapse rates after AZA withdrawal from combination therapy compared to continued combination therapy.^23^, ^24^ In their retrospective study, Mahmoud et al. also reported no increased risk of loss of response after immunomodulator withdrawal in adult IBD patients receiving anti‐TNF therapy plus immunomodulator therapy.^25^ In summary, these findings in adults and children suggest that at some point during remission, the concomitant use of an immunomodulator and a biologic is not superior to biologic monotherapy and that biologic monotherapy is tolerated by most children in remission.

Data on anti‐TNF withdrawal in children is limited. In retrospective studies, Wynads et al. and Kang et al. observed relapse rates after discontinuation of IFX while continuing immunomodulator monotherapy after combination therapy ranged from 60% to 72%. ^26^, ^27^ In summary when comparing relapse rates after withdrawal of immunomodulator versus biologic, biologic monotherapy appears being the safer and longer lasting option, which is in alignment with the results seen in our study.

Meredith et al. showed that paediatric patients with more severe disease benefit from delayed withdrawal.^4^ Our data support this, as UC patients with limited disease extent (Paris classification E1/E2) required less often therapy adjustments post‐de‐escalation than those with extensive disease (E3/E4).

In our study, we did not find any significant association between relapse and gender, age at first diagnosis or modified predictors of poor outcome.^10^ Unlike findings from adult studies by Louis et al. and Gisbert et al., lower IFX‐levels were not associated with successful de‐escalation.^18^, ^28^ While adult studies reported higher relapse rates with fecal calprotectin levels ≥300 μg/g at de‐escalation our data showed no significant differences across various calprotectin cut‐offs up to 3 months before de‐escalation (Table 3).^18^, ^28^, ^29^ This discrepancy could be due to our rather small subgroup sizes, and therefore, further research including bigger subgroups are needed.

When looking at combination therapy, it is important to distinguish those intended being de‐escalated after a certain amount of time. Of the 85 patients receiving IFX and AZA, seven received combination therapy for less than 200 days and could potentially be counted as planned de‐escalation. However, due to the small size of this subgroup, we summarized these patients in the de‐escalation group.

Limitations of our study include the exclusion of certain combination therapies, and the fact that therapy de‐escalation were not compared between the different combination therapies due to small subgroup sizes. Another limitation is the retrospective nature of the study with some missing data, for example, anti‐TNF levels before or after de‐escalation. Despite the large size and the long observation period of nearly 20 years, subgroups were rather small to achieve homogenous groups and minimize bias.

The strength of our study lies in the analysis of real‐world data from multiple centers. While our findings partially differ from adult studies and there are only few paediatric studies for comparison, it is important to note that RCTs do not represent the real‐world patient population.^30^ With this study, we capture a broad spectrum of clinical practice across diverse centers and many different patient histories, that might not have met the eligible criteria for RCTs, thereby providing valuable insights into therapy de‐escalation in everyday paediatric IBD care.

## 5 CONCLUSION

Data analysis of the large German‐Austrian multicentre registry showed that diagnosis, disease location, and what to de‐escalate to are considerable prognostic factors in the decision‐making process. We found prognostic factors that are partially consistent with those identified in adult patients. However, there is still a lack of evidence‐based pediatric data and further long‐term research is needed to identify prognostic factors for successful de‐escalation. The decision to de‐escalate should always be made in a multidisciplinary manner in the best interests of and together with the child and its family.

## CONFLICT OF INTEREST STATEMENT

Ilse Broekaert received honorary fees for presentations by Biogen and Pfizer. Jan de Laffolie received honoraria for presentations and lectures by Sanofi, Baxter, Danone, and Takeda and participated on Advisory Boards not related to IBD by Takeda, Sanofi, and Danone. The remaining authors declare no conflict of interest.

## Data Availability

The data underlying this article were provided by CEDATA‐GPGE by permission. Data will be shared on reasonable request to the corresponding author with permission of CEDATA‐GPGE. Data have been generated as part of routine clinical visits by clinicians. The manuscript, including related data, figures and tables has not been published previously and the manuscript is not under consideration elsewhere.
